# Dynamic transposable element expression during *Hydra* head regeneration

**DOI:** 10.1186/s13100-026-00406-y

**Published:** 2026-06-08

**Authors:** Aide Macias-Muñoz

**Affiliations:** Department of Ecology and Evolutionary Biology, University of California, Santa Cruz, CA 95064 USA

**Keywords:** Wnt, Jun/Fos, Apoptosis, Caspase, Metalloproteins

## Abstract

Transposable elements (TEs) contribute to genome expansions, gene duplications, and the evolution of gene regulatory networks. Recent work has highlighted the role of TE domestication in contributing to regulatory programs during animal development. Likewise, a role for TEs in regeneration has been proposed but remains understudied. *Hydra vulgaris* is a cnidarian polyp with astonishing regenerative ability. Approximately 60% of the *Hydra* genome is made up of transposable elements with a recent LINE/CR1 expansion potentially contributing to their speciation. Previous studies identified signatures of TE expression during *Hydra* regeneration, but the specific TEs involved, and their temporal expression patterns remain unknown. Here, I used publicly available RNA-sequencing data from a time course of *Hydra* head regeneration to characterize TE expression patterns. 262 TEs with dynamic temporal expression over regeneration were identified. This analysis also revealed TEs with expression patterns similar to genes involved in wound repair and tissue repatterning. Some dynamically expressed TEs were in close genomic proximity to genes involved in apoptosis, extracellular matrix remodeling, and proteins that interact with regeneration signaling pathways. This study provides a set of candidate TEs for future investigation into the mechanistic role of TEs in regeneration.

**Supplementary Information:**

The online version contains supplementary material available at 10.1186/s13100-026-00406-y.

## Introduction

Transposable elements (TEs), or mobile elements, are segments of DNA capable of moving around in the genome. They are divided into two main classes. Class I TEs, also referred to as retrotransposons, utilize a ‘copy and paste approach’ using an RNA intermediate to move around in a host genome [1]. The major retrotransposon subclasses are long terminal repeats (LTR) and non-LTR, which include LINE (Long Interspersed Nuclear Elements) and SINE (Short Interspersed Nuclear Elements). Class II TEs, or DNA transposons, move around a genome using a ‘cut and paste’ approach [1]. Major DNA transposons include terminal inverted repeats (TIR) and rolling circles (Helitrons). The movement of active TEs is an important mechanism by which genomes can evolve. TE actions can lead to gene duplications by retrotransposition, changes in gene regulatory networks, and evolution due to generating novel genetic material that can acquire new functions [2–4]. In eukaryotes, TEs have contributed to genome expansions and can be highly variable across species (reviewed in [2]). For example, in mammals, some loop anchors and CTCF sites are TE derived with species-specific distributions showing that TEs can influence 3D genome structure [5]. Yet, the movement of TEs can also lead to genome instability and deleterious heritable mutations, thus epigenetic modifications have evolved to regulate their movement and inactivate them [6, 7].

Although historically referred to as ‘junk’ or ‘selfish’ DNA, TEs have been investigated for their ‘domestication’ or ‘exaptation’ and role in phenotypes and physiological functions [8–14]. Advances in sequencing technologies now enable the assembly of chromosome-level genomes and the quantification of transposable element movement and expression, facilitating the study of TEs in biological processes, such as regeneration. Regeneration, sometimes thought of as a form of de novo development, has typically been investigated in the context of candidate conserved developmental genes [15–17]. However, some studies have inadvertently or purposefully found dynamic activation of TEs during regeneration. For example, in the sea cucumber *Holothuria glaberrima*, it was found that changes in expression of retrotransposons were associated with tissue regeneration [18]. Specifically, LTR/Gypsy elements changed in levels of expression during a time course of regeneration [18]. The high levels of expression of an element termed *Gypsy-1_Hg* during days 3–7 during regeneration drove authors to hypothesize that retrotransposon activity may play a role in regeneration [19]. In axolotls, LINE-1 (L1) was found to be expressed and hypothesized to be activated during early time points of regeneration in the blastema [20]. However, in global analyses comparing gene and retrotransposon expression in the blastema of adults and subadults, only a few elements were recovered as differentially expressed during regeneration [21]. Proper regulation of TE activity has also been recently hypothesized to be important for regeneration competency across animals [22]. Notably, a recent study found that a *Burro1* retrotransposon functions in stem cell maintenance and facilitates regeneration [23]. This TE belongs to an expansion found in Geoplanidea and Dugesiidae, land and freshwater planarians respectively, raising the question about the potential conservation or co-option of TEs as regulatory elements contributing to regeneration across animals [23].

*Hydra vulgaris* is a cnidarian polyp with incredible regenerative ability. *Hydra* has been used as a model organism for regeneration for over 280 years [24]. Many key developmental genes and transcription factors involved regeneration have been well-characterized including Wnt, Notch, Jun/Fos, and MAPK [25–31]. In *Hydra*, approximately 60% of the genome is made up of TEs [32–34]. Expansion of a LINE/CR1 element has led to an increased genome size in brown *Hydra* relative to the early branching green *Hydra* (*Hydra viridissima*) [35]. Moreover, a recent study comparing the telomere-to-telomere genomes of two laboratory strains of *Hydra vulgaris* (105 and AEP) found active TEs that are contributing to an increased genome size in AEP [36]. Interestingly, a global transcriptomic study in *Hydra* found that retrotransposon expression increases from 0 to 12 h during head regeneration [29]. Due to the timing of expression, author hypothesize a potential role in prepatterning [29]. Yet, the specific TEs that have dynamic activity and their expression profiles during *Hydra* regeneration remain unknown.

In this study, publicly available RNA-seq data from a regeneration time course of *Hydra* head regeneration was used to investigate the expression of TEs throughout the regeneration process. Differential expression analysis identified 262 TEs with dynamic expression. Clustering by expression trajectories classified these TEs into 12 groups, enabling the determination of TEs that have similar expression patterns to genes important for regeneration. Because the *Hydra* head organizer *Wnt3*, along with additional genes important for regeneration, exhibited peak expression at 12 h post-bisection, pairwise comparisons were performed comparing the 12 h regeneration time point to 0 and 48 h (early and late regeneration) to identify TEs with elevated expression at this critical time point. Overall, these analyses highlighted a potential role of DNA/hAT, DNA/Maverick, DNA/TcMar, LINE/L2, LINE/CR1, and LTR/Gypsy elements in *Hydra* regeneration.

## Methods

### Data acquisition and read mapping

The *Hydra vulgaris* 105 v3 genome was obtained from NCBI (GCA_022113875.1) [33]. RNA-seq data for regeneration time points 0 h, 2 h, 4 h, 6 h, 12 h, 24 h, 48 h were obtained from Murad et al. (GEO accession GSE127279; Table S1) [31]. The *Hydra* genome was indexed using STAR v 2.7.10b genomeGenerate with default setting and an overhang of 150 [37]. Raw RNA-seq data were trimmed and paired using trimmomatic v 0.39 ‘PE ILLUMINACLIP:NexteraPE-PE.fa:2:30:10 LEADING:3 TRAILING:3 SLIDINGWINDOW:4:15 MINLEN:36’ [38]. Paired reads were then mapped to the genome using STAR v 2.7.10b with parameters ‘–outFilterMultimapNmax 100 –winAnchorMultimapNmax 200’ to retain multi-mapped reads [37, 39].

### Transposable element identification and quantification

To identify repetitive elements in the *Hydra vulgaris* 105 v3 genome, RepeatModeler v 2.0.5 was used to create a custom repeat library [40]. The custom library was used as input for RepeatMasker to annotate the repetitive elements across the genome [41]. The RepeatMasker output file was converted into a GTF using a custom awk script that keeps the class_id annotation. TElocal utility TElocal_indexer was then used to convert the GTF into a locInd file. To quantify transposable element (TE) expression across samples, the BAM files generated by STAR were processed with TElocal to produce count tables per sample [42]. Individual count tables were concatenated using an R script to merge them by gene and TE identification.

### Differential TE expression analyses

TElocal count tables maintain gene and TE expression data. To identify differentially expressed TEs, read counts were input into R and normalized using DESeq2 v 1.48.2 [43]. A Principal Component Analysis (PCA) plot was generated to visualize sample clustering across regeneration time. To identify TEs with dynamic expression over the regeneration time course, a likelihood ratio test (LRT) including time as a factor in DESeq was used. Differentially expressed genes and TEs were then subset into clusters with similar expression trajectories. Since time point 12 h seemed to have substantial peaks of TE expression, pairwise tests were done to compare 12 h with early (0 h) and late regeneration (48 h).

### Identification of genes near candidate TEs

To determine whether regeneration-associated genes were located near repetitive elements with dynamic expression, 10 kb and 50 kb genomic windows surrounding these candidate TEs were analyzed. Candidate TEs were those with variable expression across regeneration time according to the DESeq2 analysis, specifically clusters 1, 3, 4, 7, 11, and 12, corresponding to expected trajectories for genes involved in wound healing and repatterning. Additionally, TEs that had higher expression at 12 h relative to 0 and 48 h were also considered candidates for this step. Genomic coordinates for these TEs were extracted from the GTF annotation file from RepeatMasker and converted to BED format. Using bedtools window, genes located within 10 kb and 50 kb of candidate TEs were identified and their gene descriptions and predicted products were subsequently extracted for annotation.

## Results

### Differential expression analyses

**Fig. 1:**
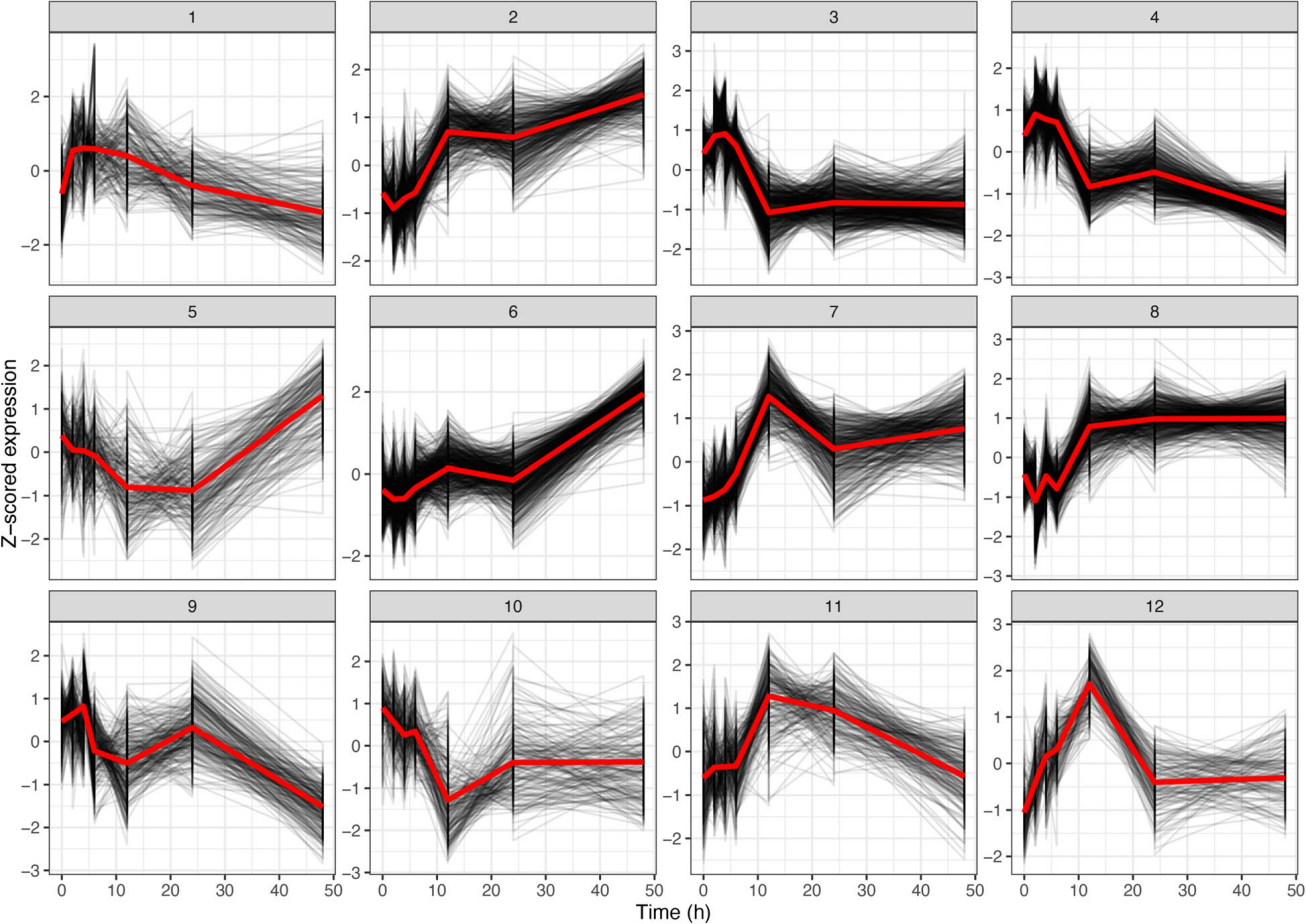
Temporal dynamic expression of genes and transposable elements during regeneration. Differentially expressed genes and TEs were grouped into clusters by similarity in expression profiles. Some clusters showed increased expression early in regeneration, some peaked around 12 h, and some showed the highest expression at 48 h post-bisection

In this study, approximately 63.82% of the *Hydra* 105 v3 genome was made up of repetitive elements, consistent with previous findings (Table S2) [32, 36]. The inclusion of TEs in differential expression analyses resulted in replicate time points clustering together with a separation of early, middle, and late regeneration, similar to analyses that excluded these elements (Fig S1) [31]. During the 48 h regeneration time course, 4,035 genes and 262 TEs showed dynamic expression (Table S3). To visualize trends in expression trajectories, these differentially expressed genes and TEs were grouped into 12 clusters by similarity in expression profiles (Fig. 1). Some clusters displayed an increase in expression early in regeneration and decreased expression after 6 h post-bisection (cluster 1, 3, and 4) (Fig. 1). Some clusters peaked in expression at 12 h post-bisection (clusters 7, 11, and 12), and some late in regeneration (clusters 2, 5, and 6) (Fig. 1).

**Fig. 2:**
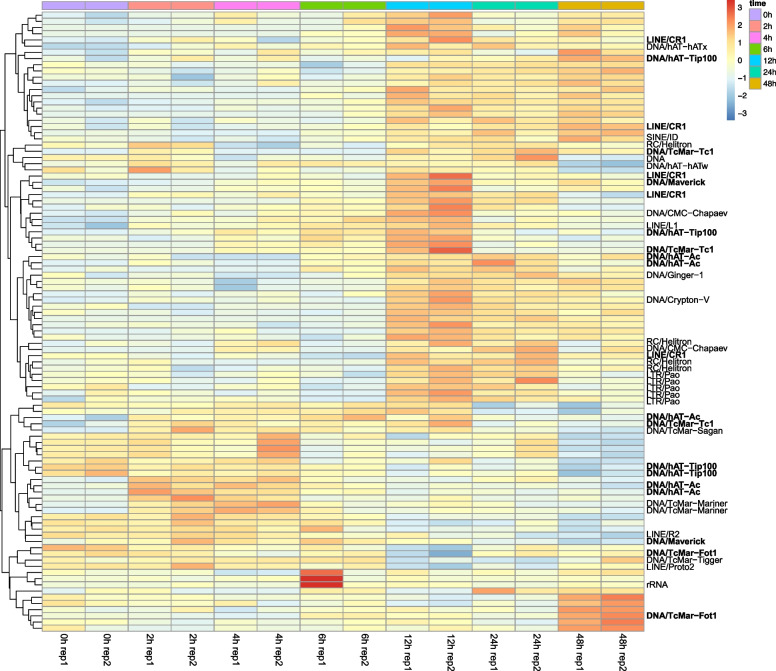
Heatmap of the top 100 differentially expressed transposable elements during regeneration. Blue is low expression and red is high expression. Labels for unknown, simple, and low complexity repeats have been removed to highlight common TEs. TE families found to be active in Kon-Nanjo et al. 2025 are in bold

Global changes in protein-coding gene expression during regeneration in *Hydr*a have been characterized previously [29–31], therefore the focus here will be TEs. Of the top differentially expressed TEs, some show peak expression around 4–6 h, 12 h, and 48 h post-bisection (Fig. 2). The TEs that peak at early time points in regeneration include DNA/hAT-Ac, DNA/hAT-Tip100, DNA/TcMar-Mariner, DNA/Maverick, and LINE/R2 (Fig. 2). TEs that peak in expression around 12 h post-bisection include LINE/CR1, DNA/Maverick, DNA/CMC-Chapaev, LINE/L1, DNA/hAT-Tip100, DNA/TcMar-Tc1, DNA/hAT-Ac, DNA/Ginger, DNA/Crypton, RC/Helitron, and LRT/Pao (Fig. 2). The TEs that peak at 48 h are mostly unknown or simple repeats with the exception of DNA/TcMar-Fot1 (Fig. 2).

### TEs with expression profiles similar to regeneration genes

To further investigate TEs with a potential role in regeneration, TEs exhibiting expression patterns similar to genes with proposed conserved functions in regeneration, and potentially being co-regulated, were identified. Jun/Fos and CREB are transcription factors involved in wound healing [30, 44]. In this study, *Jun*, *Fos*, and *creb* are all found in Cluster 1, which peaks early in regeneration (Table S4). Cluster 1 has 34 TEs with a majority being DNA elements that include DNA/TcMar-Mariner, DNA/TcMar-Tc1, DNA/Maverick, DNA/P, DNA/hAT-Ac, DNA/CMC-EnSpm, DNA/TcMar-Sagan, DNA/hAT-hATw, and DNA/Sola-2. Cluster 1 also contains two LINE elements, LINE/CR1 and LINE/L2, and one LTR/Gypsy element (Table S4). *Wnt3*, *Wnt7*, and *Wntless* are Wnt family genes associated with regeneration in *Hydra* [26, 29, 30]. In this study, these genes along with *EGR*, a potentially conserved regeneration transcription factor [44, 45], are found in Cluster 7, which peaks at 12 h post-bisection. Cluster 7 contains 40 TEs including seven DNA elements, four LINE elements, and one LTR element. The annotated TEs include DNA/PIF-ISL2EU, DNA/Maverick (2), DNA/TcMar-Fot1, DNA/CMC-Chapaev, DNA/Crypton-V, DNA/Ginger-1, LINE/L2, LINE/CR1 (3), and LTR/Gypsy (Table S4).

### TEs upregulated at 12 h post-bisection

**Fig. 3:**
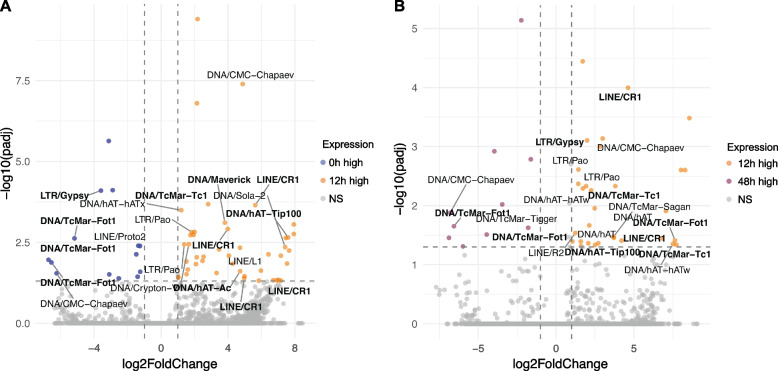
Transposable elements highly expressed at 12 h post-bisection. **A** Volcano plot of TE expression at 12 h (gold) relative to 0 h (blue). **B** Volcano plot of TE expression at 12 h (gold) versus 48 h (pink). Non-gray colored circles represent significantly upregulated TEs with adjusted p-values > 0.05 and logFC > 2, horizontal dashed line represents adjusted p-value of 0.05, and vertical dashed lines represent logFC of 1. Unknown, simple, and low complexity repeats are not labeled. TE families found to be active in Kon-Nanjo et al. 2025 are in bold

*Wnt3*, the *Hydra* head organizer gene, peaks in expression at 12 h post head bisection [31]. As noted in the top 100 differentially expressed TEs and cluster trajectories, many TEs peak around 12 h post-bisection. To further characterize TEs that may be active at this stage of regeneration, pairwise comparisons were performed between 12 and 0 h, and between 12 h and late regeneration (48 h). 815 genes and 48 repetitive elements were upregulated at 12 h compared to 0 h (Table S5). These TEs included DNA/Sola-2, DNA/CMC-Chapaev, DNA/CMC-EnSpm, DNA/TcMar-Fot1, DNA/TcMar-Tc1, DNA/hAT-Ac, DNA/hAT-Tip100, DNA/hAT-hATx, DNA/Crypton-V, DNA/PIF-ISL2EU, LINE/L1, LINE/L2, LINE/CR1, RC/Helitron, LTR/Gypsy, LTR/Pao (TableS5; Fig. 3). Between 12 and 48 h, 341 genes and 35 TEs were upregulated at 12 h. These TEs included DNA/hAT, DNA/TcMar-Fot1, DNA/TcMar-Sagan, DNA/TcMar-Tc1, DNA/CMC-Chapaev, DNA/hAT-hATm, DNA/hAT-hATw, DNA/hAT-Tip100, DNA/CMC-EnSpm, LINE/CR1, LINE/R2, LTR/Gypsy, and LTR/Pao. Overall, these complementary analyses highlight shared trends in activation patterns and identify TEs that may play a role in *Hydra* head regeneration.

### Genomic context of dynamic TEs

Identification of TEs that have similar expression to genes important for regenerative functions hints at potential co-regulation however it is unclear where they may be found in a genomic or cellular context. To determine whether any of these TEs were located near genes important for regeneration, 10 kb and 50 kb windows surrounding each TE were examined to identify nearby protein-coding genes. This approach identified genes with functions in transcriptional regulation, extracellular matrix remodeling, and cytoskeletal functions (Table S7; Table S8). Some direct candidates for functions in regeneration included genes involved in apoptosis and cell maintenance. There were three *caspase* genes located near DNA elements from the hAT-hATx and CMC-EnSpm families that were upregulated at 12 h, a *THAP domain-containing protein 1-like* gene located near a DNA/P element upregulated early in regeneration, and a *Fem-1 homolog* near a DNA/Maverick element that peaked at 12 h (Table S7; Table S8). Interestingly, there was an *apoptosis-resistant E3 ubiquitin protein ligase 1* and *RING finger and CHY zinc finger domain-containing protein* near an unknown TE with expression early in regeneration (Table S8). Metalloproteinases were also identified near dynamic TEs including *matrix metalloproteinase-24* near an unknown TE expressed early in regeneration, two *zinc metalloproteinases* near an unknown and LINE/CR1 element also expressed early in regeneration, and two *A disintegrin and metalloproteinase with thrombospondin motifs 7* (ADAMTS7) near DNA elements CMC-EnSpm and TcMar-Tc1 that peaked in expression at 12 h (Table S8).

Additional candidates for regeneration included genes associated with signaling, developmental regulation, and tissue remodeling (Table S8). For example, a *slit homolog* was identified near an unknown TE upregulated early in regeneration and a *von Willebrand factor D and EGF domain-containing protein* near a DNA/hAT-Ac element that was also upregulated early in regeneration. Similarly, a *CREB-regulated transcription co-activator* was located near a DNA/hAT-Ac element upregulated early in regeneration. An *activin receptor* was identified near an unknown TE that peaked at 12 h. A *MAPK-interacting protein* was near an unknown TE that peaked at 12 h, an *myb-like protein* near an RC/Helitron element that peaked at 12 h, and multiple *ras-related proteins* were found located near LINE/L1, DNA/Ginger, DNA/TCMar-Mariner, and unknown TEs. Lastly, two *fibroblast growth factor receptor 1* (*FGFR*) genes were located near unknown TEs that peaked at 12 h. Results from this flanking region analysis suggest that TEs may play a role in regeneration through cellular maintenance, remodeling, and stress-response functions.

## Discussion

A surprising finding of animal regeneration transcriptomic studies has been the identification of TEs that appear to be transcribed after injury [18, 20, 29]. These findings have led researchers to propose that TEs become active as a response to amputation stress or have a role in regeneration [19, 20]. Whether TEs have a direct or indirect role in regeneration across animals is unknown. While the role of TEs in animal development has gained attention, the role of TEs in regeneration remains understudied. Specifically, large scale investigations focusing on characterizing TEs and their expression patterns during regeneration are largely lacking. This study used bioinformatics tools tailored to the analysis of TEs to characterize their expression during *Hydra* head regeneration. 262 TEs were found to have dynamic expression with some being similar expression profiles to genes important for injury response and patterning.

### Most Hydra ‘active TEs’ have dynamic expression during regeneration

A recent study identified ‘active TEs’ through a comparative genomic analysis of *Hydra vulgaris* strains 105 and AEP [36]. Researchers identified 14 TE families that contributed to major expansions in AEP and confirmed their transcriptional expression with Iso-Seq [36]. These active TEs included DNA/hAT-Ac, DNA/hAT-Charlie, DNA/hAT-hATm, DNA/hAT-Tip100, DNA/TcMar-Tc1, DNA/TcMar-Fot1, DNA/Maverick, DNA/Sola-2, DNA/MULE-MuDR, DNA/CMC-EnSpm, DNA/CMC-Transib, LINE/CR1, LINE/2, and LTR/Gypsy. These active TE families have been highlighted in our study by being bolded across figures. A majority of these TEs were recovered as having dynamic expression during regeneration. With the exception of DNA/hAT-Charlie, DNA/MULE-MuDR, and DNA/CMC-Transib, members of the active TE families display dynamic expression, with different elements within the same family peaking either during early regeneration and/or at 12 h post-bisection. The dynamic expression of active TEs suggests some potential models of TE mechanisms. Firstly, TE dynamic expression could be explained by global chromatin opening during regeneration similar to embryonic development [12, 14]. If this is the case, TEs may be passively transcribed with no role in regeneration. Alternatively, active TE expression may mirror that of genes important for regeneration if they share regulatory sequences. A third model could be that this activity may indicate the co-option of TE-derived sequences for regulatory functions during regeneration. The overlap between dynamically expressed TEs and active TEs in *Hydra* is consistent with prior reports and supports their ongoing transcriptional activity [29, 36].

### Expression of retrotransposons associated with regeneration in other animals

TEs that have been highlighted as potentially having a role in animal regeneration are LTR/Gypsy and LINE/L1 [18–20]. One LINE/L1 element appears in the top 100 differentially expressed TEs (Fig. 2). This element (rnd-1_copy586266:rnd-1:family-17:LINE/L1) clusters with TEs that peak in expression around 12 h post-bisection (Fig. 2) and is upregulated in the pair-wise comparison between 12 and 0 h post-bisection (Fig. 3A). For LTR/Gypsy, six copies appear to have dynamic expression in our analysis. The LTR/Gypsy upregulated at 12 versus 0 h (rnd-5_copy303046:rnd-5:family-4745:LTR/Gypsy) is in cluster 12 which increases in expression after injury, peaks at 12 h, and then decreases. The LTR/Gypsy upregulated at 12 versus 48 h (rnd-4_copy288095:rnd-4:family-5945:LTR/Gypsy) is in cluster 11. The remaining LTR/Gypsy elements are in clusters 1, 7, 10 and 11. These results indicate that multiple LTR/Gypsy elements peak in expression at 12 h post-bisection. However, with the exception of rnd-5_copy303046 that is within 50 kb of an RNA helicase, most Gypsy elements are not in close genomic proximity to genes important for regeneration. One LTR/Gypsy element expanded in some planarians (*Burro1*) has a demonstrated function in regeneration by having acquired a B-IAP region consisting of a RING (really interesting new gene) and BIR (Baculovirus IAP repeat) domain that enhances regenerative capacity by safeguarding stem cells against caspase-mediated apoptosis [23]. Interestingly, this study recovered a similar pattern with an Unknown TE element located near an intronless RING domain gene and an *apoptosis-resistant E3 ubiquitin protein ligase*. This element was upregulated early in regeneration and a search for its sequence in the Dfam database classified it as a LINE-dependent_Retroposon; SINE; U-RNA_Promoter. Since *Burro1* is planarian-specific, it was not expected that a homolog would be found in *Hydra*, nevertheless the presence of many dynamic TEs near apoptosis-related genes is a notable finding. This suggests a potential recurring association between TE activity and cell survival pathways during regeneration.

### Dynamic TEs near genes associated with injury response and cellular maintenance

To date, one of the most comprehensive studies of TE function in animal regeneration is the investigation of *Burro1*, which localized expression of this element to stem cells and provided functional validations for its role in their survival. In this study, dynamically expressed TEs were found near genes associated with regeneration-related signaling pathways, including CREB-, MAPK-, and FGFR-associated components. Dynamic TEs were also found near genes important for transcriptional regulation and processing including multiple zinc finger proteins and spliceosomal RNAs (Table S7; Table S8). An interesting result was the finding of genes associated with apopotosis and extracellular remodeling. Three caspase genes were in close proximity to dynamic TEs. Caspases have been demonstrated to play a role in apoptosis in *Hydra* during regeneration, through the activation of Wnt signaling [28, 30, 46]. Similarly, metalloproteins and regulation of the extracellular matrix remodeling are essential for proper *Hydra* head regeneration [47–49]. The proximity of dynamically expressed TEs to genes involved in apoptosis and extracellular matrix remodeling may indicate that TE activity may be linked to tissue repair following injury, although direct regulatory relationships remain to be experimentally validated.

### Limitations of current research

The study of TEs in *Hydra* regeneration has now been advanced by studies describing a dynamic expression of TEs [29], identification of a brown-hydra specific expansion of LINE/CR1 elements [35], and the recent discovery of active TE families driving genome evolution in the two commonly used lab *Hydra* strains [36]. Our study complements this work by taking on a locus-focused investigation to characterize the expression of TEs during regeneration. A limitation of the field lies in the pervasive challenge of accurately assembling and mapping repetitive sequences. A family-level approach is less sensitive to mis-matched reads, but also less precise in detecting a potential functional co-option of TEs. A locus-level approach is promising in this area but subject to biases or ambiguity in read mapping. Additionally, an open question is that of co-expression at the cellular level. Some TE families have been found to have cell type specific expression in *Hydra*, with some being expressed in stem cells [36]. In this bulk RNA-seq approach, some markers for *Hydra* stem cell genes showed generally steady expression over regeneration time, with some peaking at 24 h post bisection and some showed increased expression from 2 to 24 h (Fig S2). Investigation of the single-cell expression patterns of these TEs would provide additional insight. However, the current *Hydra* single-cell atlas was generated for the AEP strain. Although the strains belong to the same species, their TEs landscapes differ, which may make it difficult to characterize and compare them across strains [36, 50]. Moreover, current single-cell atlases are generated from short-read sequencing, which would still be complicated by the TEs repetitive nature. An important next step in the field will be to study TE expression using single-cell long-read sequencing data, which would enable detection of locus-level candidate TEs and regeneration genes at a single-cell resolution.

Together, these genome-wide results support and extend previous findings that TEs exhibit dynamic transcriptional activity during regeneration. A focus on *Hydra*, a well-studied model organism for regeneration, enables the identification of TEs that peak at important time points during the regenerative process and may be co-expressed with wound response and patterning genes. An interesting finding is that of individual TEs being transcribed in a variable and dynamic manner, which may not fully be explained by global chromatin opening. While transcriptional activity does not directly indicate active TEs undergoing transposition, this study does highlight a set of TEs for further investigation into the molecular mechanisms by which they may affect regenerative ability. A future direction of this research includes the functional testing of active TEs that appear in these complementary analyses.

## Conclusions

The role of TEs in regeneration is an understudied and promising research subject. This study corroborates that TEs have temporal dynamic expression during regeneration and highlights candidate TEs for future investigation. In *Hydra*, 262 elements were found to have dynamic expression over a time course of head regeneration. Differential expression analyses identified TEs that appear co-expressed with genes important for wound response and tissue repatterning. Some of these candidate TEs include members of TE families that have been previously identified as being transcriptionally active and expressed stem cells in *Hydra* AEP. In addition, dynamic TEs are located in close genomic proximity to genes associated with cell survival, extracellular matrix remodeling, and regeneration-related signaling pathways. These findings shed light on the differential expression of TEs during regeneration and provide a set of candidate TEs to investigate their functional roles and potential conservation across regenerative species.

## Supplementary Information

Supplementary Material 1: Figure S1. RNA-seq PCA plot

Supplementary Material 2: Figure S2. Wnt3, vasa, piwi, and nanos gene expression trajectories

Supplementary Material 3: Table S1. RNA-seq data Accession Table

Supplementary Material 4: Table S2. Hydra 105 v3 RepeatMasker output

Supplementary Material 5: Table S3. DESeq Likelihood Ratio Test (LRT) results

Supplementary Material 6: Table S4. Genes and TEs in Clusters 1-12

Supplementary Material 7: Table S5. Genes and TEs upregulated at 12 versus 0 hours post-bisection

Supplementary Material 8: Table S6. Genes and TEs upregulated at 12 versus 48 hours post-bisection

Supplementary Material 9: Table S7. Genes within 10 kb of dynamically expressed repetitive elements

Supplementary Material 10: Table S8. Genes within 50 kb of dynamically expressed repetitive elements

## Data Availability

Raw sequencing data was obtained from NCBI GEO accession GSE127279. The counts table and analysis scripts are available in zenodo 10.5281/zenodo.20237191. The outputs of RepeatModler and RepeatMasker are on figshare 10.6084/m9.figshare.32316876.

## Abbreviations

TE: Transposable elements
LTR: Long terminal repeats
LINE: Long interspersed nuclear elements
SINE: Short interspersed nuclear elements
RNA-seq: RNA-sequencing
PCA: Principal Component Analysis
LRT: Likelihood ration test

## References

[1] Miller W j., Capy P. Mobile genetic elements as natural tools for genome evolution. In: Miller WJ, Capy P, editors. Methods in Molecular Biology. Humana Press; 2004. 10.1385/1-59259-755-6:001 15020798

[2] Feschotte C, Pritham EJ. DNA transposons and the evolution of eukaryotic genomes. Annu. Rev. Genet. 2007. p. 331–68. 10.1146/annurev.genet.40.110405.090448 10.1146/annurev.genet.40.110405.090448 PMC2167627 18076328

[3] Feschotte C. Transposable elements and the evolution of regulatory networks. Nat Rev Genet. 2008;9:397–405. 10.1038/nrg2337. 18368054 10.1038/nrg2337 PMC2596197

[4] Chuong EB, Elde NC, Feschotte C. Regulatory activities of transposable elements: from conflicts to benefits. Nat Rev Genet. 2017;18:71–86. 10.1038/nrg.2016.139. 27867194 10.1038/nrg.2016.139 PMC5498291

[5] Choudhary MNK, Quaid K, Xing X, Schmidt H, Wang T. Widespread contribution of transposable elements to the rewiring of mammalian 3D genomes. Nat Commun. 2023;14. 10.1038/s41467-023-36364-9 10.1038/s41467-023-36364-9 PMC9902604 36746940

[6] Platt RN, Vandewege MW, Ray DA. Mammalian transposable elements and their impacts on genome evolution. Chromosome Research. Springer Netherlands; 2018. p. 25–43. 10.1007/s10577-017-9570-z 10.1007/s10577-017-9570-z PMC5857283 29392473

[7] Kohlrausch FB, Berteli TS, Wang F, Navarro PA, Keefe DL. Control of LINE-1 expression maintains genome integrity in germline and early embryo development. Reprod Sci. 2022;29:328–40. 10.1007/s43032-021-00461-1. 33481218 10.1007/s43032-021-00461-1

[8] Bourque G. Transposable elements in gene regulation and in the evolution of vertebrate genomes. Curr Opin Genet Dev. 2009;19:607–12. 10.1016/j.gde.2009.10.013. 19914058 10.1016/j.gde.2009.10.013

[9] Garcia-Perez JL, Widmann TJ, Adams IR. The impact of transposable elements on mammalian development. Development (Cambridge). Company of Biologists Ltd; 2016. p. 4101–14. 10.1242/dev.132639 10.1242/dev.132639 PMC5830075 27875251

[10] Bourque G, Burns KH, Gehring M, Gorbunova V, Seluanov A, Hammell M, et al. Ten things you should know about transposable elements. Genome Biol. 2018;19:1–12. 10.1186/s13059-018-1577-z. 30454069 10.1186/s13059-018-1577-z PMC6240941

[11] Bejerano G, Lowe CB, Ahituv N, King B, Siepel A, Salama SR, et al. A distal enhancer and an ultraconserved exon are derived from a novel retroposon. Nature. 2006;441:87–90. 10.1038/nature04696. 16625209 10.1038/nature04696

[12] Gerdes P, Richardson SR, Mager DL, Faulkner GJ. Transposable elements in the mammalian embryo: pioneers surviving through stealth and service. Genome Biol. 2016;17:1–17. 10.1186/s13059-016-0965-5. 27161170 10.1186/s13059-016-0965-5 PMC4862087

[13] Hackett JA, Kobayashi T, Dietmann S, Surani MA. Activation of lineage regulators and transposable elements across a pluripotent spectrum. Stem Cell Reports. 2017;8:1645–58. 10.1016/j.stemcr.2017.05.014. 28591649 10.1016/j.stemcr.2017.05.014 PMC5470235

[14] Low Y, Tan DEK, Hu Z, Tan SYX, Tee WW. Transposable Element Dynamics and Regulation during Zygotic Genome Activation in Mammalian Embryos and Embryonic Stem Cell Model Systems. Stem Cells Int. 2021. 10.1155/2021/1624669 10.1155/2021/1624669 PMC8536462 34691189

[15] Brockes JP, Kumar A. Comparative aspects of animal regeneration. Annu Rev Cell Dev Biol. 2008;24:525–49. 10.1146/annurev.cellbio.24.110707.175336. 18598212 10.1146/annurev.cellbio.24.110707.175336

[16] Bely AE, Nyberg KG. Evolution of animal regeneration: re-emergence of a field. Trends Ecol Evol. 2010;25:161–70. 10.1016/j.tree.2009.08.005. 19800144 10.1016/j.tree.2009.08.005

[17] Aztekin C, Storer MA. To regenerate or not to regenerate: Vertebrate model organisms of regeneration-competency and -incompetency. Wound Repair and Regeneration. 2022;1–13s. 10.1111/wrr.13000 10.1111/wrr.13000 PMC7613846 35192230

[18] Mashanov VS, Zueva OR, García-Arrarás JE. Posttraumatic regeneration involves differential expression of long terminal repeat (LTR) retrotransposons. Dev Dyn. 2012;241:1625–36. 10.1002/dvdy.23844. 22911496 10.1002/dvdy.23844

[19] Mashanov VS, Zueva OR, García-Arrarás JE. Retrotransposons in animal regeneration: overlooked components of the regenerative machinery? Mob Genet Elements. 2012;2:244–7. 23550104 10.4161/mge.22644 PMC3575433

[20] Zhu W, Kuo D, Nathanson J, Satoh A, Pao GM, Yeo GW, et al. Retrotransposon long interspersed nucleotide element-1 (LINE-1) is activated during salamander limb regeneration. Dev Growth Differ. 2012;54:673–85. 10.1111/j.1440-169X.2012.01368.x. 22913491 10.1111/j.1440-169X.2012.01368.x PMC3529008

[21] Ruiz-Pérez S, Alcaraz N, Torres-Arciga K, Ocampo-Cervantes JA, Cervera A, Castro-Hernández C, et al. Retrotransposon expression is upregulated in adulthood and suppressed during regeneration of the limb in the Axolotl (*Ambystoma mexicanum*). Advanced Biology. 2025. 10.1002/adbi.202400502. John Wiley and Sons Inc. 40443108 10.1002/adbi.202400502 PMC12517315

[22] Angileri KM, Bagia NA, Feschotte C. Transposon control as a checkpoint for tissue regeneration. Development. 2022;149. 10.1242/dev.191957 10.1242/dev.191957 PMC10655923 36440631

[23] Lee HL, Poulet A, Allikka Parambil S, van Wolfswinkel JC. Host-transposon mutualism supports regeneration in planarians. Dev Cell. 2026. 10.1016/j.devcel.2026.03.001. Cell Press;. 41916287 10.1016/j.devcel.2026.03.001 PMC13047482

[24] Galliot B. Hydra, a fruitful model system for 270 years. Int J Dev Biol. 2012;56:411–23. 10.1387/ijdb.120086bg. 22855328 10.1387/ijdb.120086bg

[25] Chera S, Ghila L, Dobretz K, Wenger Y, Bauer C, Buzgariu W, et al. Apoptotic cells provide an unexpected source of Wnt3 signaling to drive Hydra head regeneration. Dev Cell. 2009;17:279–89. 10.1016/j.devcel.2009.07.014. (**Elsevier Ltd;**). 19686688 10.1016/j.devcel.2009.07.014

[26] Nakamura Y, Tsiairis CD, Ozbek S, Holstein TW. Autoregulatory and repressive inputs localize Hydra Wnt3 to the head organizer. Proc Natl Acad Sci U S A. 2011;108:9137–42. 10.1073/pnas.1018109108. 21576458 10.1073/pnas.1018109108 PMC3107325

[27] Moneer J, Siebert S, Krebs S, Cazet J, Prexl A, Pan Q, et al. Differential gene regulation in DAPT-treated Hydra reveals candidate direct Notch signalling targets. J Cell Sci. 2021. 10.1242/jcs.258768. 34346482 10.1242/jcs.258768 PMC8353520

[28] Chera S, Ghila L, Wenger Y, Galliot B. Injury-induced activation of the MAPK/CREB pathway triggers apoptosis-induced compensatory proliferation in Hydra head regeneration. Dev Growth Differ. 2011;53:186–201. 10.1111/j.1440-169X.2011.01250.x. 21338345 10.1111/j.1440-169X.2011.01250.x

[29] Petersen HO, Höger SK, Looso M, Lengfeld T, Kuhn A, Warnken U, et al. A comprehensive transcriptomic and proteomic analysis of Hydra head regeneration. Mol Biol Evol. 2015;32:1928–47. 10.1093/molbev/msv079. 25841488 10.1093/molbev/msv079 PMC4833066

[30] Cazet J, Cho A, Juliano C. Generic injuries are sufficient to induce ectopic Wnt organizers in Hydra. Elife. 2021;10:1–31. 10.7554/eLife.60562. 10.7554/eLife.60562 PMC8049744 33779545

[31] Murad R, Macias-Muñoz A, Wong A, Ma X, Mortazavi A. Coordinated gene expression and chromatin regulation during Hydra head regeneration. Genome Biol Evol. 2021;13:1–17. 10.1093/gbe/evab221. 10.1093/gbe/evab221 PMC8651858 34877597

[32] Chapman JA, Kirkness EF, Simakov O, Hampson SE, Mitros T, Weinmaier T, et al. The dynamic genome of Hydra. Nature. 2010;464:592–6. 10.1038/nature08830. 20228792 10.1038/nature08830 PMC4479502

[33] Simakov O, Bredeson J, Berkoff K, Marletaz F, Mitros T, Schultz DT, et al. Deeply conserved synteny and the evolution of metazoan chromosomes. Sci Adv. 2022;8(5):eabi5884. 35108053 10.1126/sciadv.abi5884 PMC8809688

[34] Cazet JF, Siebert S, Little HM, Bertemes P, Primack AS, Ladurner P, et al. A chromosome-scale epigenetic map of the Hydra genome reveals conserved regulators of cell state. Genome Res. 2023;33:283–98. 10.1101/gr.277040.122. 36639202 10.1101/gr.277040.122 PMC10069465

[35] Wong WY, Simakov O, Bridge DM, Cartwright P, Bellantuono AJ, Kuhn A, et al. Expansion of a single transposable element family is associated with genome-size increase and radiation in the genus Hydra. Proc Natl Acad Sci U S A. 2019;116:22915–7. 10.1073/pnas.1910106116. 31659034 10.1073/pnas.1910106116 PMC6859323

[36] Kon-Nanjo K, Kon T, Yu TCTK, Rodriguez-Terrones D, Falcon F, Martínez DE, et al. The dynamic genomes of Hydra and the anciently active repeat complement of animal chromosomes. Genome Biol. 2025;26. 10.1186/s13059-025-03653-z 10.1186/s13059-025-03653-z PMC12220107 40597406

[37] Dobin A, Davis CA, Schlesinger F, Drenkow J, Zaleski C, Jha S, et al. STAR: ultrafast universal RNA-seq aligner. Bioinformatics. 2013;29:15–21. 10.1093/bioinformatics/bts635. 23104886 10.1093/bioinformatics/bts635 PMC3530905

[38] Bolger AM, Lohse M, Usadel B. Trimmomatic: a flexible trimmer for Illumina sequence data. Bioinformatics. 2014;30:2114–20. 10.1093/bioinformatics/btu170. 24695404 10.1093/bioinformatics/btu170 PMC4103590

[39] Rodríguez-Quiroz R, Valdebenito-Maturana B. SoloTE for improved analysis of transposable elements in single-cell RNA-Seq data using locus-specific expression. Commun Biol. Nature Research; 2022;5. 10.1038/s42003-022-04020-5 10.1038/s42003-022-04020-5 PMC9537157 36202992

[40] Flynn JM, Hubley R, Goubert C, Rosen J, Clark AG, Feschotte C, et al. RepeatModeler2 for automated genomic discovery of transposable element families. PNAS BioMed Central Ltd. 2020;117:9451–7. 10.1186/s13059-018-1577-z. 10.1073/pnas.1921046117 PMC7196820 32300014

[41] Smit AHR& GP. RepeatMasker Open-4.0. http://www.repeatmasker.org.

[42] Jin Y, Tam OH, Paniagua E, Hammell M. TEtranscripts: a package for including transposable elements in differential expression analysis of RNA-seq datasets. Bioinformatics. 2015;31:3593–9. 10.1093/bioinformatics/btv422. 26206304 10.1093/bioinformatics/btv422 PMC4757950

[43] Love MI, Huber W, Anders S. Moderated estimation of fold change and dispersion for RNA-seq data with DESeq2. Genome Biol. 2014;15:1–21. 10.1186/s13059-014-0550-8. 10.1186/s13059-014-0550-8 PMC4302049 25516281

[44] Srivastava M. Beyond casual resemblance: rigorous frameworks for comparing regeneration across species. Annu Rev Cell Dev Biol. 2021;37:415–40. 10.1146/annurev-cellbio-120319-114716. 34288710 10.1146/annurev-cellbio-120319-114716

[45] Gehrke AR, Neverett E, Luo YJ, Brandt A, Ricci L, Hulett RE, et al. Acoel genome reveals the regulatory landscape of whole-body regeneration. Science. 2019;363. 10.1126/science.aau6173 10.1126/science.aau6173 30872491

[46] Cikala M, Wilm B, Hobmayer E, Böttger A, David CN. Identification of caspases and apoptosis in the simple metazoan Hydra . 1999. http://biomednet.com/elecref/0960982200900959 10.1016/s0960-9822(99)80423-0 10508589

[47] Yan L, Leontovich A, Fei K, Sarras MP. *Hydra* metalloproteinase 1: a secreted astacin metalloproteinase whose apical axis expression is differentially regulated during head regeneration. Dev Biol. 2000;219:115–28. 10.1006/dbio.1999.9568. 10677259 10.1006/dbio.1999.9568

[48] Sarras MP Jr, Yan LI, Leontovich A, Zhang JS, Sarras MP. Structure, expression, and developmental function of early diver-gent forms of metalloproteinases in *Hydra*. Cell Res. 2002;12(3–4):163–76. 10.1038/sj.cr.7290123. 12296376 10.1038/sj.cr.7290123

[49] Cox BD, Mah J, Perez A, Juliano CE. Extracellular matrix remodeling supports *Hydra vulgaris* head regeneration and stem cell invasion. bioRxiv. 2025. 10.1101/2025.11.07.687297.41659553

[50] Mays A, Cabrera F, Macias-Munoz A. Comparative analysis of transposable elements in jellyfish and hydroid species (Cnidaria: Medusozoa). bioRxiv. 2026. 10.64898/2026.04.17.719288

