# Supplementary material for: Dynamic transposable element expression during Hydra head regeneration
Source: Mob DNA. 2026 Jun 8;17:25. doi: 10.1186/s13100-026-00406-y (PMC13465017; doi:10.1186/s13100-026-00406-y)
Supplement: Supplementary file 1 [file 13100_2026_406_MOESM10_ESM.pdf]

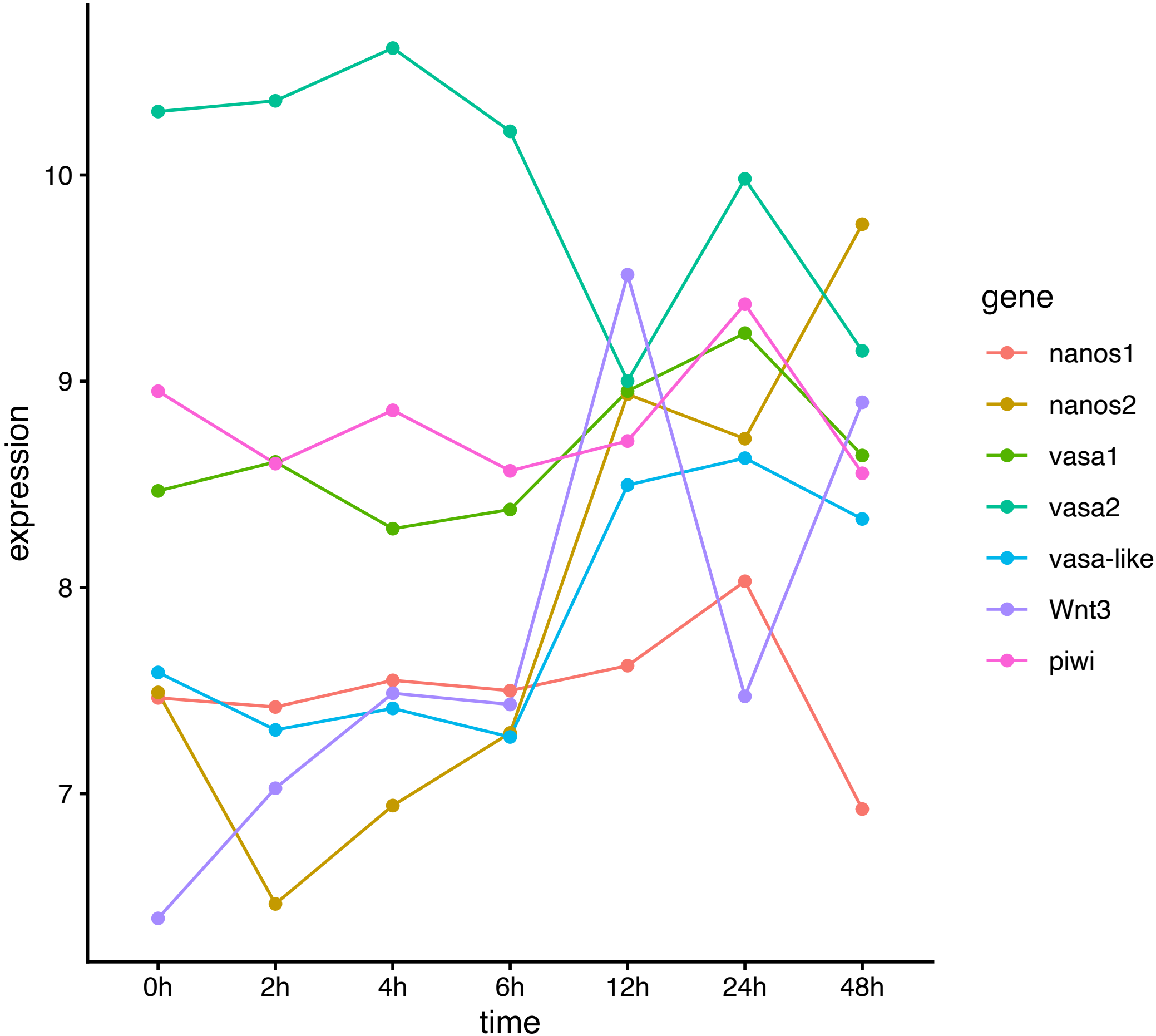

10
9
8
7
expression
0h
2h
4h
6h
12h
24h
48h
time
gene
nanos1
nanos2
vasa1
vasa2
vasa-like
Wnt3
piwi
