# Supplementary material for: Dynamic transposable element expression during Hydra head regeneration
Source: Mob DNA. 2026 Jun 8;17:25. doi: 10.1186/s13100-026-00406-y (PMC13465017; doi:10.1186/s13100-026-00406-y)
Supplement: Supplementary file 2 [file 13100_2026_406_MOESM9_ESM.pdf]

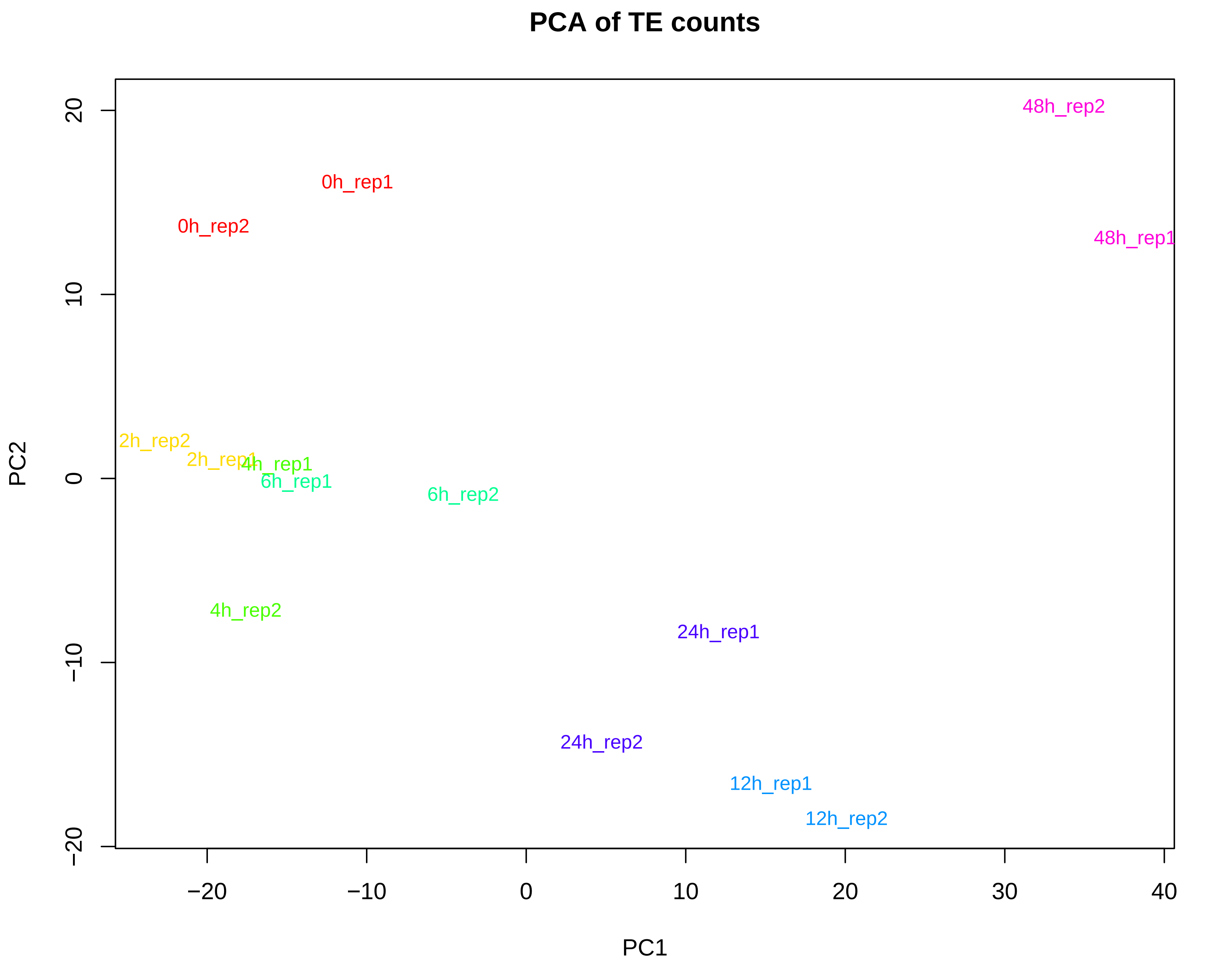
PCA of TE counts
PC1
PC2
0h_rep1
0h_rep2
48h_rep2
48h_rep1
2h_rep2
2h_rep1
4h_rep1
6h_rep1
6h_rep2
4h_rep2
24h_rep1
24h_rep2
12h_rep1
12h_rep2
−20
−10
0
10
20
30
40
20
10
0
−10
−20
